# Complete chloroplast genome sequencing of Job’s tears (*Coix* L.): genome structure, comparative analysis, and phylogenetic relationships

**DOI:** 10.1080/23802359.2021.1911704

**Published:** 2021-04-15

**Authors:** Xiang-Dong Li, Hong Pan, Xiu-Juan Lu, Xin-Yuan Wei, Ming Shi, Ping Lu

**Affiliations:** a Southwest Guizhou Institute of Karst Regional Development, Xingyi, Guizhou, China; b Adlay of Engineering Technical Research Centre in Guizhou, Xingyi, Guizhou, China; c Institute of Crop Sciences, Chinese Academy of Agricultural Sciences, Beijing, China

**Keywords:** Chloroplast, genome structure, phylogeny, adlay

## Abstract

Job’s tears, also known as adlay, is a valuable plant that has commonly been used in traditional Chinese medicine, as well as an edible food. Due to the lack of knowledge of its genetics and gaps in its evolutionary analysis, breeding of adlay has been hindered. Here, we report five complete chloroplast genomes of various species and varieties in the genus by Illumina sequencing, while their genome structure, comparative analysis, and phylogenetic relationships were conducted. Genome sizes ranged from 140,860 to 140,864 bp in length, GC contents were 38.43%, and genome architecture was of a typical quadripartite structure. We annotated 82～83 protein-coding genes and 46～47 non-coding RNA genes in each genome and they functionally associated with self-replication, photosynthesis, cytochrome synthesis and other unknown functions. Three codons that encoded tryptophan, arginine and leucine were used frequently at rates of 41.42, 37.98, and 32.28% respectively. The preferred codons consistently ended with A or T. A total of 146 simple sequence repeats (SSR), 9 insertions and deletions (InDels) and 143 single nucleotide polymorphisms (SNPs) were observed among genomes. The InDel and SNP variations were mostly distributed in intergenic regions. It confirmed that *Coix*, *Sorghum*, *Saccharum, Zea*, *Tripsacum* and *Saccharum* were closely genera and the genetic distance of *Sorghum* to *Coix* was closer t*han Zea to Coix.* These results give us more insight into the evolution of *Coix* in a wide range of evolutionary studies.

## 1. Introduction

Job’s tears, also known as adlay, belongs to the *Coix* L. genus in the Gramineae family. This plant has been used as medicine and as a source of food (Arora 1977; Yang et al. 2011). Also, adlay has potential as forage for animals in agriculture due to its high protein content and large biomass (Zhou et al. 2019). Modern pharmacological studies have shown that extracts from adlay may have diverse pharmacological effects, including anticancer, antioxidant, and anti-inflammatory properties, regulation of fat metabolism, and more (Yang et al. 2011; Huang et al. 2012).

The chloroplast is the photosynthetic organelle of most green plants, where both developmental processes and secondary metabolic activities take place (Wicke et al. 2011). Though it primarily functions as an energy factory, the chloroplast also facilitates coordination of gene expression between organelles and the nuclear genome (Woodson and Chory 2008). The chloroplast genome whose genes were transcribed polycistronic in clusters, is considered to have originated from an ancestral endosymbiotic cyanobacteria (Kanno and Hirai 1993; Yoon et al. 2006). Unlike chromosome genes, chloroplast DNA is inherited matrilineally and has a relatively moderate nucleotide substitution rate owing to lower genetic selection pressure. Therefore, chloroplast genetic information can be a useful tool to research plant phylogeny and evolution (Zhou et al. 2016; Zhang et al. 2017), species identification and taxonomy (Wu et al. 2010; Kuang et al. 2011). Some gene fragments such as *matK*, *rbcL*, *rpoC1,* and *trnA-psb* were successfully used as DNA barcoding for Chinese herbal species recognition and molecular identification. Otherwise, the chloroplast is potential to be used vectors for genetic engineering.

Traditionally, the genus *Coix* included about 10 species or varieties in the world, while it was classified with 6_～_12 species or varieties by different Chinese taxonomic reports (Zhuang et al. 1994; Li and Qin 1995; Lu and Zuo 1996). To date, its taxonomy remains controversial, recent efforts have provided insight into the evolutionary relationships and domestication of *Coix* species with the sequencing and assembly of chromosome genomes of *C. lacryma-jobi* and *C. aquatica* (Guo et al. 2020; Liu et al. 2020). In addition, the complete chloroplast genome of *C. lacryma-jobi* was obtained by long-fragment PCR amplification and its molecular evolutionary relationships to cereal relatives were also discussed (Leseberg and Duvall 2009; Kang et al. 2018).

Despite the important dietary and medicinal values of *Coix*, genomic research of the chloroplast is very limited; its evolutionary and taxonomic relationships with other grass crops still need to be elucidated. Here, we report chloroplast genomes of five species or varieties and their genome structures and phylogenetic relationships.

## 2. Materials and methods

### 2.1. Plant materials and DNA sequencing

Five purported species (or varieties) of *Coix*, *C. puellarum* Balansa (Xiaozhuyiyi, XZ), *C. stenocarpa Balansa* (Zhaiguoyiyi, ZG), *C. chinensis* var. *formosana* (Ohwi) L. Liu.(Taiwanyiyi, TW), *C . lacryma-jobi* var*. maxima* Makino(Nianzhuyiyi, NZ), and *C. chinensis* var. *chinesis* Tod(Yimi, YM), were selected for study. Individuals of each taxon were self-pollinated for more than six generations before being sampled for genome sequencing and assembly (Table S1). Young seedlings with 4_～_6 leaves were used to extract total DNA. Approximately 5 g of fresh leaves was harvested for DNA isolation using extraction method (Chen et al. 2011). After isolating the DNA, 1 μg of purified DNA was fragmented to construct short-insert libraries according to the manufacturer’s instructions and then sequenced on the Illumina Hiseq 4000 (Borgstrom et al. 2011).

### 2.2. Genome assembly and annotation

Prior to assembly, Illumina raw reads were filtered. This filtering step was performed in order to remove reads with adaptors, reads showing a quality score below 20(Q < 20), reads containing a percentage of uncalled based (“N” characters) equal or greater than 10% and duplicated sequences. The chloroplast genome was reconstructed using the Illumina Hiseq data, and the following three steps were used to assemble chloroplast genomes. First, we assembled the genome framework from the Illumina data using SPAdes v3.10.1 (Dmitry et al. 2016). Second, we verified the assembly and completed the circle characteristic of the chloroplast genome while any potential filling gaps in the sequence. Third, clean reads were mapped to the assembled chloroplast genome to correct bases and record any insertions and deletions.

Chloroplast genes were annotated using homology alignments and *de novo* prediction, and Evidence Modeler v1.1.1 (Haas 2008) was used to integrate gene predictions. Transfer RNA (tRNA) and ribosomal RNA (rRNA) genes were predicted by tRNAscan-SE (Lowe and Eddy 1997) and rRNAmmer 1.2 (Lagesen et al. 2007). A whole-genome BLAST search (E-value ≤ 1e^−5^, minimal alignment length percentage ≥40%; Altschul et al. 1990) of the chloroplast data was performed against five databases. They are KEGG (Kyoto Encyclopedia of Genes and Genomes, Kanehisa 1997; Kanehisa et al. 2004, 2006), COG (Clusters of Orthologous Groups, Tatusov et al. 1997; Tatusov et al. 2003), NR (Non-Redundant Protein Database), Swiss-Prot (Magrane 2011), and GO (Gene Ontology, Ashburner et al. 2000). A map of the chloroplast genome was drawn using Organellar Genome DRAW v 1.2 (Lohse et al. 2007).

### 2.3. Sequence analysis

Besides our five assembled genomes, another three chloroplast genomes of *C. lacryma-jobi* (FJ261955.1, MH558672.1, KY596160.1) were also analyzed . Relative synonymous codon usage (RSCU) values were obtained using MEGA5.2 (Tamura et al. 2011). We used REPuter (http://bibiserv.techfak.uni-bielefeld.de/reputer/) to seek long repeats (forward, palindrome, complement and reverse sequences).The MISA Perl script was also used to detect simple sequence repeats (SSRs) with a motif size of one to six nucleotides and thresholds of eight, four, four, three, three, and three, respectively.

### 2.4. Comparative genome analysis

Genome size and organization of chloroplasts were compared, and the differences of the IR border of eight adlay chloroplast genomes were analyzed. The *C. puellarum* (ZI000287) chloroplast genome was used as a reference to aligned with the other seven genomes using mVISTA software. Insertion/deletion and SNP loci were detected by MUMmer and LASTZ software.

### 2.5. Phylogenetic analysis

The whole-genome alignment of the chloroplast genomes of eight species in the genus *Coix* and other gramineous plants, including *Zea mays* (NC_001666.2, X86563.2), *Zea luxurians* (NC_030301.1), *Sorghum bicolor* (EF115542.1, NC_008602.1), *Tripsacum dactyloides* (NC_037087.1, MG386499.1), *Saccharum officinarum* (NC_006084), *Oryza nivara* (NC_005973), *Oryza sativa* ssp. *indica* (JN861110.1), *O. sativa* ssp. *japonica* (KM088017.1), *Brachypodium distachyon* (NC_011032), *Agrostis stolonifera* (NC_008591), *Triticum aestivum* (NC_002762), *Hordeum vulgare* (NC_008590), *Festuca arundinacea* (NC_011713), *Lolium perenne* (NC_009950), *Bambusa oldhamii* (NC_012927), and *Dendrocalamus latiflorus* (NC_013088) that were available in the NCBI Organelle Genome Resources database, was used to build a phylogenetic tree of all these taxa using the maximum likelihood (ML) method. Details of genomes used above are listed in Table S2.

## 3. Results

### 3.1. Chloroplast genome features

The DNA sequences of five adlay genomes were acquired using the Illumina platform. We obtained 3886_～_8137 Mb of Illumina raw data and 3,647_～_7,792 Mb of clean data from each species(variety) with a high quality scores of 96.84_～_98.66%(Q20). The complete chloroplast genomes ranged from 140,860 to 140,864 bp in size and all contained the same GC content of 38.43%. The genomes have a typical quadripartite architecture that consists of a pair of IRs (22,757 bp), a SSC region (12,521_～_12,522 bp) and a LSC region (82,825_～_82,828 bp), similar to complete chloroplast genomes of other adlay varieties (Leseberg and Duvall 2009; Kang et al. 2018) (Table S3).

### 3.2. Gene annotation and codon usage bias

By whole-genome blast to KEEG, COG, NK, Swiss-Prot and GO databases, we annotated 82_～_83 protein-coding genes and 46_～_47 non-coding RNA genes, including 39 tRNAs, two *rrn*4.5, two *rrn*5, two *rrn*16 and two *rrn*23 genes (Tables S4 and S5). The protein-coding genes occupied 40.78%_～_41.30% and non-coding genes occupied 8.64% of the whole genome. Like those of other green plants, the protein-coding genes could be classified into groups of small and large subunits of ribosomes (*rps*11, *rps*12, *rps*14, *rps*15, *rps*16, *rps*18, *rps*19, *rps*2, *rps*3, *rps*4, *rps*7, *rps*8, *rpl*14, *rpl*16, *rpl*2, *rpl*20, *rpl*22, *rpl*23, *rpl*32, *rpl*33, *rpl*36), RNA polymerase subunits (*rpo*A, *rpo*B, *rpo*C1, *rpo*C2), NADH dehydrogenases (*ndh*A, *ndh*B, *ndh*C, *ndh*E, *ndh*F, *ndh*G, *ndh*H, *ndh*I, *ndh*J), photosynthetic-related genes of the Photosystem I and II complexes, the ATP synthase and protease complex, the large subunit of rubisco, maturase, envelope membrane protein, C-type cytochrome synthesis and other unknown functional genes of *ycf*3 and *ycf*4. We also determined that seven tRNA genes(*trn*K-UUU, *trn*G-UCC, *trn*T-GGU, *trn*L-UAA, *trn*V-UAC, *trn*I-GAU, *trn*A-UGC) and nine protein-coding genes(*rps*16, *atp*F, *rps*12, *pet*B, *pet*D, *rpl*16, *rpl*2, *ndh*A, *ndh*B) received one intron and the protein-coding gene *ycf*3 received two introns.

Codon usage was calculated for the protein-coding genes present in the five adlay genomes to provide essential information in the evolution of the genus *Coix*. We determined that tryptophan, arginine and leucine codons were frequently used in a ratio of 41.42, 37.98, and 32.28%, respectively. In contrast, the codons that encoded cysteine had the lowest usage ratio of 2.20%. Additionally, it was supported by relative synonymous codon usage (RSCU) values that a total of 29 codons were biased used and 27 preferential codons ended with A or a T in the third nucleotide position. No codon bias was observed for the proline(CCA), methionine(ATG), tryptophan(TGG) amino acids (Table S6).

### 3.3. Repetitive sequence features

In total, 146 SSRs were found by the MISA analysis. There were 128, 5, 2, 9, 1 and 1, mono-, di-, tri-, tetra-, penta- and hexa- repeats, respectively, among the eight adlay genomes we examined, with a distributed ratio of 87.67%, 2.74%, 1.37%, 6.16%, 0.68%, and 0.68%, respectively. Additionally, the mononucleotide A/T was most commonly found in the adlay genomes at a high ratio of 84.25% (Table S7). These new SSRs will be potentially useful for population studies in the *Coix* L. genus, possibly in combination with other informative nuclear genome SSRs.

### 3.4. Inverted repeat contraction and expansion

**Figure 1.**
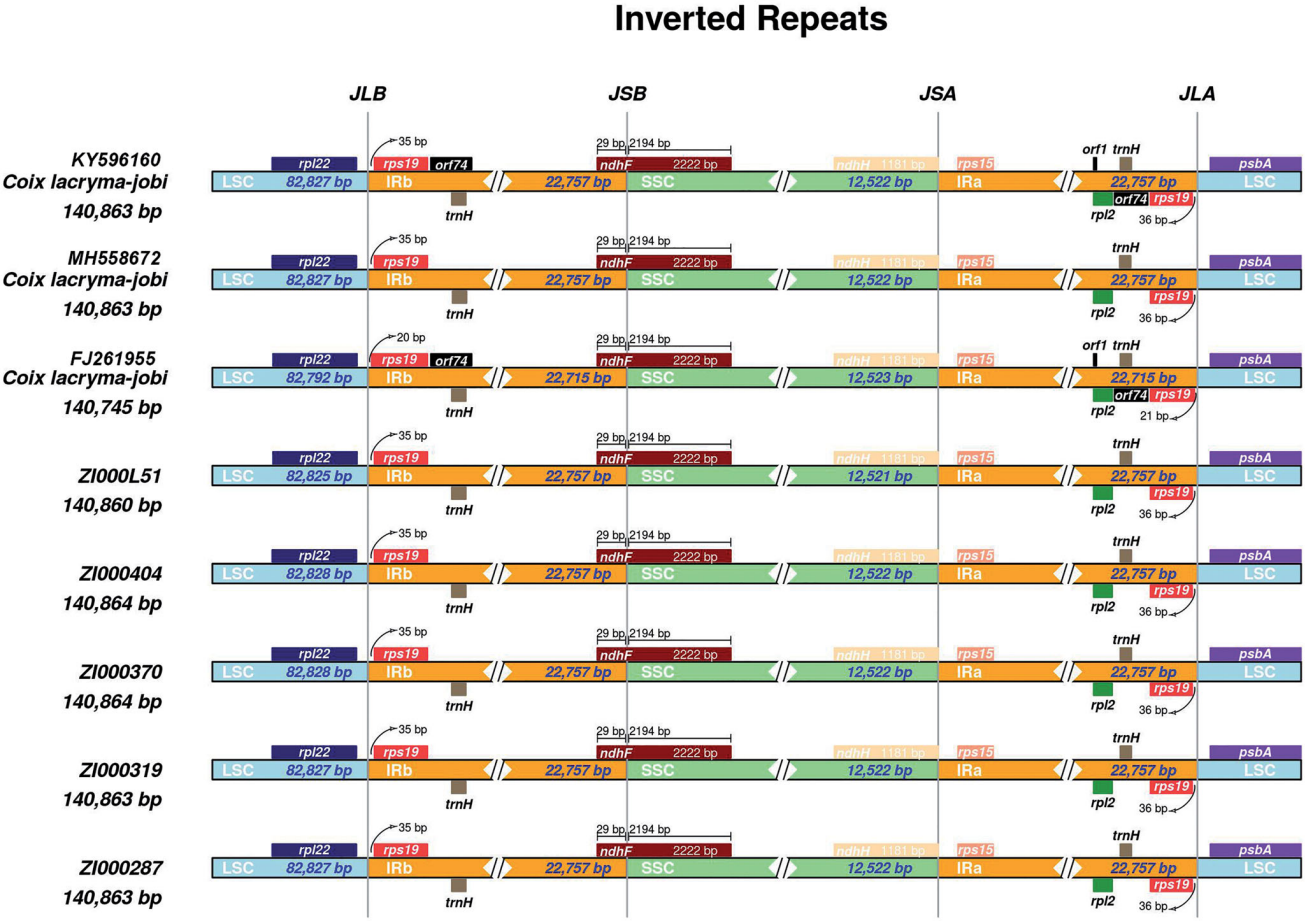
Comparison of the junction between inverted repeat region (IR), large single copy-region (LSC) and small single copy-region (SSC) of chloroplast genome among eight Coix species (varieties).

The junctions between the IR and LSC/SSC regions among the eight adlay chloroplast genomes were compared (Figure 1). The eight adlay chloroplast genomes were highly conserved; there were very slight discrepancies among the genomes. The LSC/IR and SSC/IR borders were sharply marginated in all complete chloroplast genomes, and only the genome of *C. lacryma-jobi* (FJ261955.1) shrunk slightly. Gene *rps*19 in the LSC region extended from 20_～_35 bp into the IRa region and 21_～_36 bp into the IRb region. Although *orf*74 and *orf*1 were present in the IR region of two *C. lacryma-jobi* (FJ261955.1 and KY596160.1) genomes, no obvious IR regions expansion were observed. Commonly, the *ndh*F gene overlapped by 29 bp into IRb among the eight genomes. In addition, there is just slight differences in IR boundary regions that *orf1* and *orf74* genes were only observed in KY596160 and FJ261955 genomes.

### 3.5. Chloroplast genome comparisons

**Figure 2.**
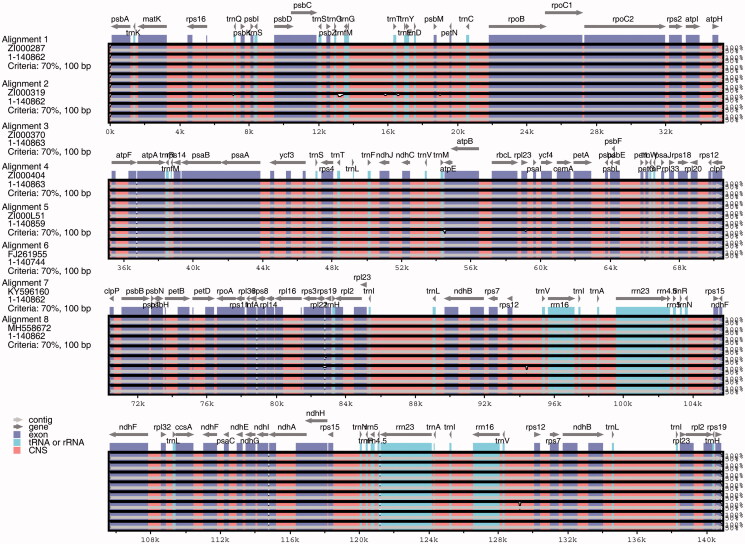
Comparison of eight chloroplast genomes of adlay. *Note*: Gray arrows and thick black lines above the alignment indicate gene orientation. Purple bars represent exons, pink bars represent conserved non-coding sequences (CNS), and blue bars represent mRNA. The y-axis represents the percentage identity (shown: 50–100%).

**Figure 3.**
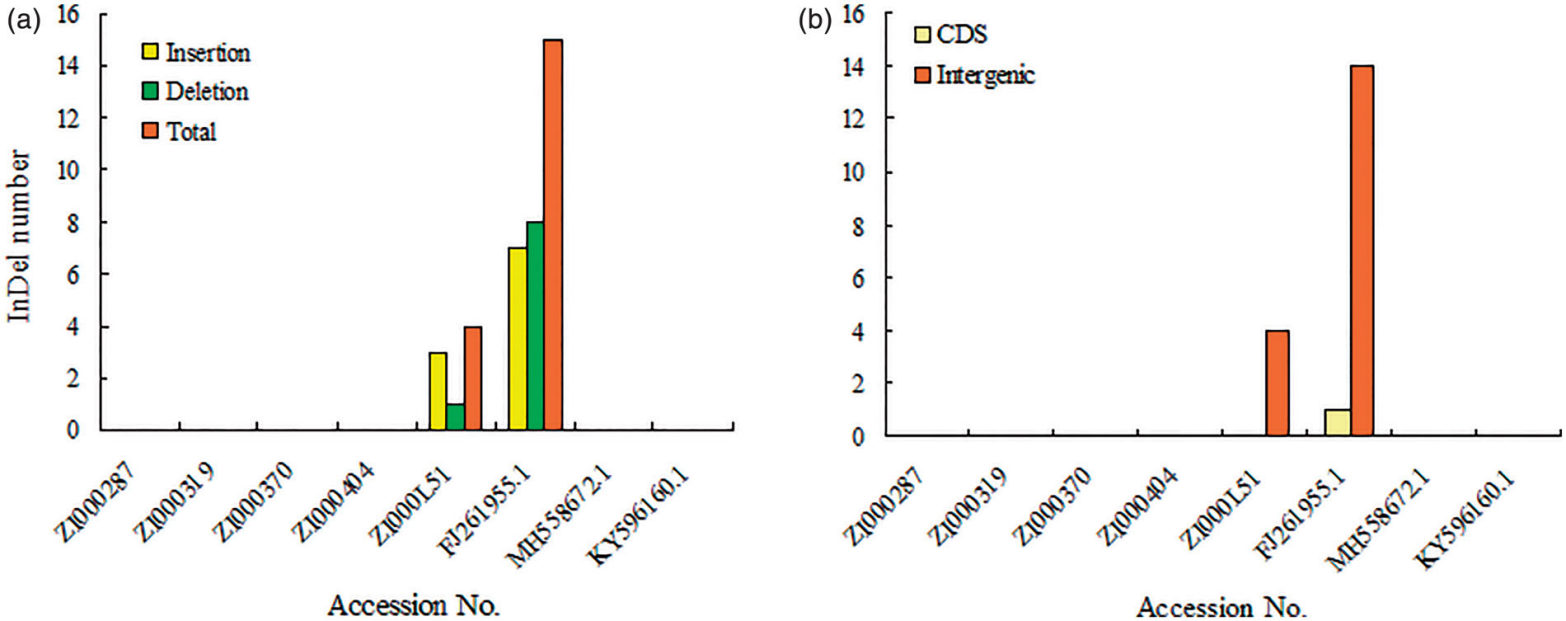
Statistics of InDel variations.

The results of the genome alignment indicate that genomes of the eight *Coix* taxa were conserved and showed a high degree of synteny and gene order (Figure 2). Nevertheless, we observed genetic divergence in both gene introns and intergenic spacers. With ZI000287 as the reference genome, a total of 19 InDels and 143 SNPs were observed among genomes (Figure 3). However, only 1 InDel and 10 SNP mutations were located in the introns of genes, including *psb*N, *rpo*A, *rpo*B, *rpo*C1, *rps*2, *rps*19, *atp*B, *rpl*22, *rpl*23, and *ndh*F. The residues were intergenic, revealing that mutations occurs more frequently in intergenic than intragenic regions. The LSC and SSC regions also had more variation than IRs. The variations we uncovered are areas that need further investigation into the phylogenetic development and evolutionary relationships of the genus *Coix*.

### 3.6. Genomic phylogeny

**Figure 4.**
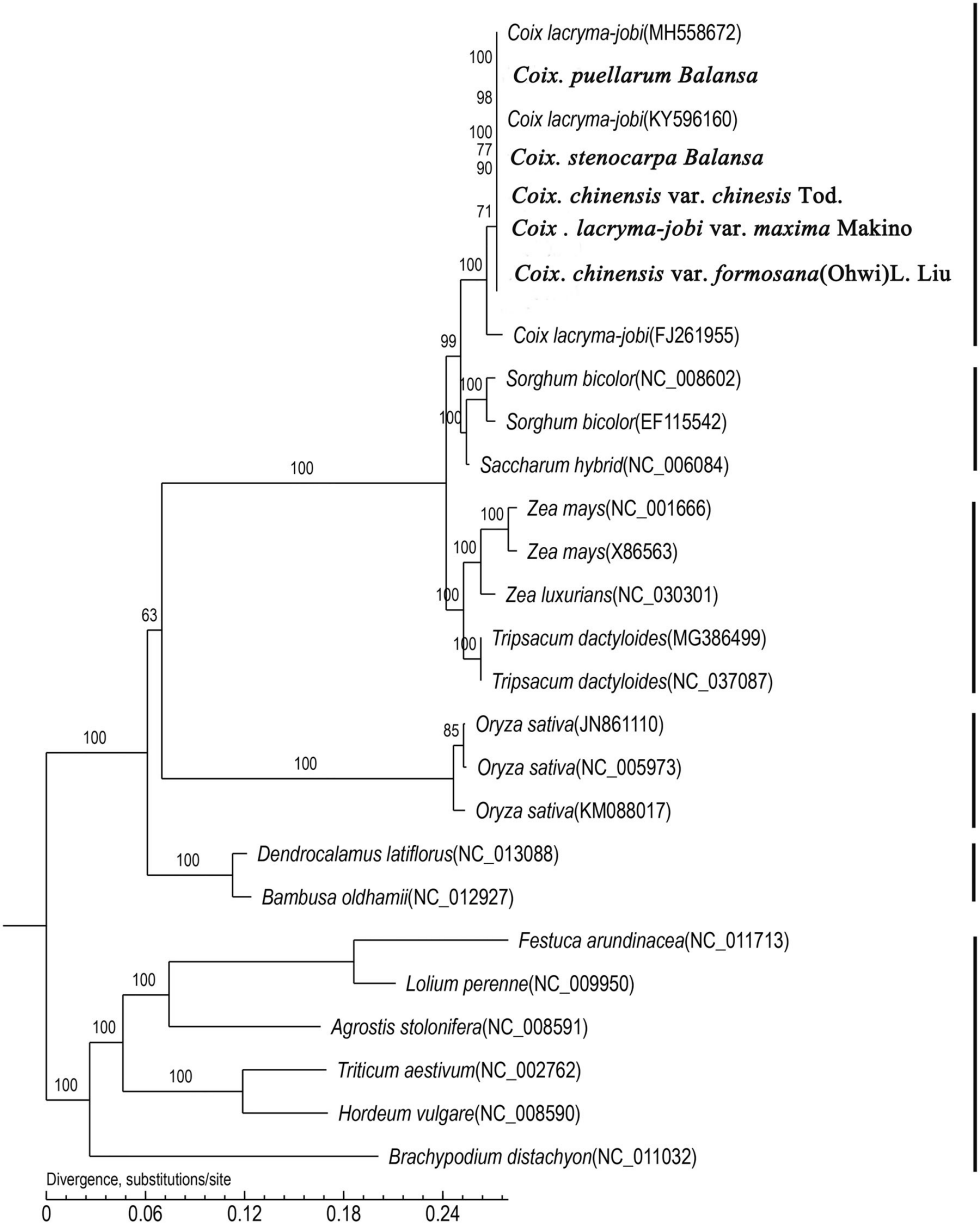
Maximum likelihood (ML) phylogenetic tree. *Note*: The phylogenetic tree including eight *Coix* species(varieties) and nineteen other species based on concatenated sequences from all chloroplast genomes.

A ML phylogenetic analysis was performed on the whole twenty-seven genome sequences. The phylogeny tree shows that the eight adlay taxa clustered together and had two sister clades that included *Sorghum bicolor*, a *Saccharum* hybrid, *Zea mays*, *Zea luxurians* and *Tripsacum dactyloides*. The analysis suggests that *Sorghum bicolor* was more closely related to *Coix* than *Zea mays* or *Zea luxurians*. In addition, *Zea mays* and *Zea luxurians* appear to be close relatives of *Tripsacum dactyloides*, and *Saccharum* was also closely related to *Sorghum* (Figure 4). Overall, we have evidence that *Coix*, *Sorghum*, *Saccharum, Zea*, *Tripsacum* and *Saccharum* are closely related genera with *Sorghum*, while the *Sorghum* genus had the shortest genetic distance to *Coi*x. These results give us more insight into the evolution of *Coix* in a wide range of evolutionary studies.

## 4. Discussion

### 4.1. Chloroplast comparative genomics

Here, we report five complete chloroplast genome sequences and structural information of adlay (*Coix* spp.) from the following five taxa *C. puellarum* Balansa, *C. stenocarpa Balansa*, *C. lacryma-jobi* var*. maxima* Makino, *C. chinensis* var. *chinesis* Tod., *C. chinensis var. formosana(Ohwi)*L. Liu. The genomes ranged in size from 140,860 to 140,864 bp, exhibited quadripartite architecture and had a relatively high GC content of 38.43%. These genome features were very similar to each other, as well as other plants (Saski et al. 2007; Leseberg and Duvall 2009; Kang et al. 2018). We also obtained high quality scores of 96.84_～_98.66% (Q20) from the raw data and successfully ascertained entire genomes without any sequence gaps. Also, in methodology angle, we firstly extracted total DNA for sequencing and subsequently divided the chloroplast sequence data by bioinformatics operations; this strategy was used to avoid the mix nuclear DNA and simplified working process compared with the traditional method (Leseberg and Duvall 2009). Despite the strong similarities among the genomes, differences were still present. Simple sequence repeats are usually found in chloroplast genomes, and can be used as molecular markers of genetic diversity and for evolutionary research (Huang et al. 2014). In this study, we found a total 146 SSRs in all eight genomes with an abundance of the mononucleotide A/T, which was geared to other plants (Tanvi et al 2017; Shen et al. 2018; Zhang et al. 2018). The genome analysis showed that most InDels and SNPs were distributed intergenically and we were able to annotate many genes, genes that may be invaluable for genus or species identification as in herbs (Ma et al. 2018).

### 4.2. Coix phylogeny in Maydeae

Based on complete plastid genome sequences, it have provided valuable insights into relationships among and within plant genera in many cereal plants, such as *Hordeum*, *Sorghum* or *Camellia* (Saski et al. 2007; Huang et al. 2014). In traditional taxonomy, the genera *Coix*, *Zea* and *Tripsacum* belong to the grass tribe Maydeae, while *Sorghum* and *Saccharum* belong to the tribe Andropogoneae. Thus in the past, because of their similar plant phenotypes, *Zea mays* was considered the closest relative to adlay. However, genetic information refutes that relationship and instead indicates that *Sorghum* and *Saccharum* are more similar to *Coix* than *Zea*, which is supported by the karyotyping and repetitive sequence analysis reported by Cai et al. (2014). We suspect that a recent whole-genome duplication event occurred in *Coix*, independent of *Zea*, and then the original species of *C. aquatica* and *S. bicolor* diverged_～_10.0 million year ago (Liu et al. 2020; Guo et al. 2020). Our chloroplast genome phylogeny confirmed supports the previous conclusions as well.

## 5. Conclusion

This paper reports five complete chloroplast genomes that were sequenced from five taxa of the genus *Coix* (*C. puellarum* Balansa, *C. stenocarpa Balansa*, *C. chinensis var. formosana(Ohwi)*L. Liu., *C . lacryma-jobi var. maxima* Makino and *C. chinensis var. chinesis* Tod. We determined similar genome drafts, structures, gene compositions and repetitive sequences among the different adlay species or varieties. Genomic comparisons also revealed that genomes were highly conserved, while the genomic regions that varied, such as SSR, InDel and SNP loci, provide opportunities to fully exploit genetic technology such as DNA barcoding or molecular markers for species identification. Furthermore, our phylogenetic analysis still reveals that *Coix*, *Sorghum*, *Saccharum, Zea*, and *Tripsacum* were closely related genera with *Sorghum* having the shorter genetic distance to *Coix* than *Zea*.

## Data Availability

The five genome sequences assembly has been deposited into NCBI GenBank under project ID: MT471102 (*C. puellarum* Balansa)(https://www.ncbi.nlm.nih.gov/nuccore/MT471102), MT471094 (*C. stenocarpa Balansa*)(https://www.ncbi.nlm.nih.gov/nuccore/MT471094), MT471095 (*C.lacryma-jobi var. maxima* Makino) (https://www.ncbi.nlm.nih.gov/nuccore/MT471095), MT471096 (*C. chinensisvar. chinesis* Tod.) (https://www.ncbi.nlm.nih.gov/nuccore/MT471096), MT471101 (*C. chinensis var. formosana(Ohwi)*L. Liu) (https://www.ncbi.nlm.nih.gov/nuccore/MT471101).
